# A model for HIV/AIDS pandemic with optimal control

**DOI:** 10.1063/1.4915640

**Published:** 2015-05-15

**Authors:** Amiru Sule, Farah Aini Abdullah

**Affiliations:** Pusat Pengajian Sains Matematik, Universiti Sains Malaysia, 11800 USM, Penang, Malaysia; Universiti Malaysia Perlis, Perlis, Malaysia; Universiti Malaysia Perlis, Perlis, Malaysia; Universiti Malaysia Perlis, Perlis, Malaysia; Universiti Malaysia Perlis, Perlis, Malaysia; Universiti Malaysia Perlis, Perlis, Malaysia

## Abstract

Human immunodeficiency virus and acquired immune deficiency syndrome (HIV/AIDS) is pandemic. It has affected nearly 60 million people since the detection of the disease in 1981 to date. In this paper basic deterministic HIV/AIDS model with mass action incidence function are developed. Stability analysis is carried out. And the disease free equilibrium of the basic model was found to be locally asymptotically stable whenever the threshold parameter (RO) value is less than one, and unstable otherwise. The model is extended by introducing two optimal control strategies namely, CD4 counts and treatment for the infective using optimal control theory. Numerical simulation was carried out in order to illustrate the analytic results.

## INTRODUCTION

Human immunodeficiency virus and Acquired immunodeficiency syndrome (HIV/AIDS), is a global pandemic. It affects all regions of the world. Back in 1981 when the first case of HIV was detected nearly 75 million people were infected so far. Likewise more than 36 million people died of HIV/AIDS related incidences [1]. Globally 35.3 million people were living with HIV/AIDS as at 2012. Majority of them were found in the low and middle income countries. And about 25 million infected were found in sub Saharan Africa. That is 71% of the global infection. Additionally the report indicates that each day HIV/AIDS pandemic causes approximately 6300 new infection and about 4300 deaths in 2012 were recorded that means, there were about 2.3 million new infections in the year [2], against 2.5 million new infections in 2011, also, about 1.6 million people died of aids in 2012. Against 1.7 million people that died of the disease in 2011.this indicate the number of people contracting the disease is reducing, largely due to public awareness/ campaign. Also the number of people dying of the disease is reducing as a result of availability of life saving treatment for infected people, compared to the previous years. There are two types of HIV; HIV-1 and HIV-2, but HIV-1 is more virulent and is the cause of the majority of the HIV infection globally. Because it can easily be transmitted HIV-2 is only found in the West central African sub region. And is less transmitted [3].

In the recent times, some epidemiological models have used optimal control, some of which focus on HIV/AIDS and other infectious diseases. Among which include [4-11].The optimal control efforts are carried out to limit the spread of the disease. And in some cases to prevent the emergence of drug resistance, health workers use a test that counts the number of CD4+ cells in cubic millimetre of blood. Normal CD4+ count of a healthy HIV negative adult varies, but it is usually between 600 and 1200 cells/mm$^3$. Even though, it may be lower in some people. Mostly people infected with HIV find that their CD4+ counts fall over time and it happens at a variable rate, but the CD4+ counts can still be stable for long period.

Hence, it is useful for HIV infected people to have their CD4+ count measured regularly so as, (1) to monitor their immune system that help them whether and when to start taking HIV treatment, and other treatment to prevent infection. (2) And also, to monitor the effectiveness of HIV treatment. It is recommended that every HIV infected individuals start taking life saving drugs when the CD4+ cell count is around 350cell/mm$^3$ [12]. Even after the commencement of the treatment i.e lifesaving drugs for HIV/AIDS patients, CD4+ counts, is still needed so that, to ensure the effectiveness of the drugs, otherwise the combination of the drugs should be change. In this study the stages of infections as categorized by world health organization and centre for disease control are considered [13]. The stages are asymptomatic stage, symptomatic stage and aids stage.

The main objective of this paper, Is to develop a mathematical model for human interaction with the aim of carrying out the stability analysis. Using two optimal control strategies namely, CD4+ counts,, and treatment for the infective on combating the spread of HIV/AIDS. The organization of the paper is as follows: In section 2, description of the general model formulation and stability analysis is given. In section 3, the optimal control formulation with two optimal control strategies is presented. Section 4 contains the simulation results and illustrating the results of the dynamics. Lastly the conclusion is given in section 5.

*International Conference on Mathematics, Engineering and Industrial Applications 2014 (ICoMEIA 2014)*
AIP Conf. Proc. 1660, 050007-1–050007-10; doi: 10.1063/1.4915640
© 2015 AIP Publishing LLC 978-0-7354-1304-7/$30.00

## MODEL FORMULATION

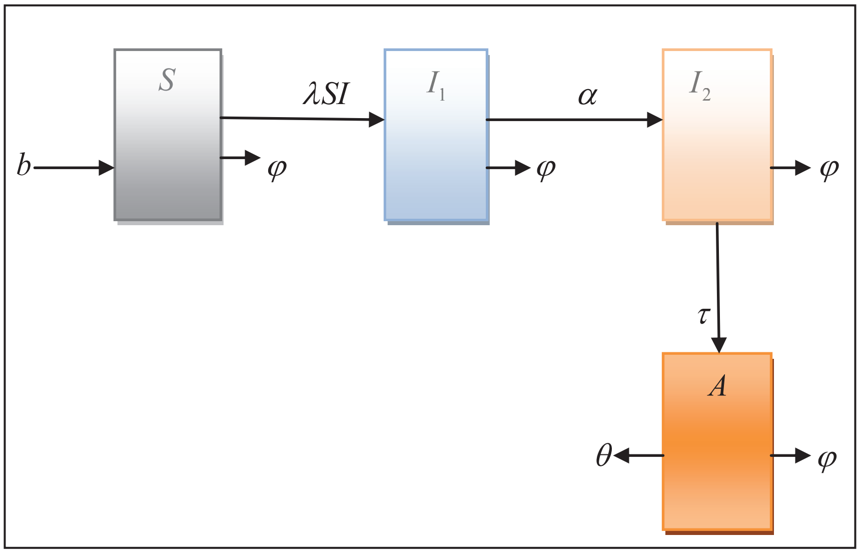

**FIGURE 1.** Transfer diagram of HIV/AIDS.

The total human population at time t denoted by N (t), is subdivided into four classes of susceptible $(S(t))$. These are sexually active individuals that are free from infection, but they are prone to infection as they interact with infected humans. Asymptomatic class ( $I_1(t)$ ), these are infected humans, but does not show any symptom of infection. Symptomatic class $(I_2(t))$, these are set of infected individuals in which the symptoms of infection are manifested. And finally Aids class $(A(t))$, these are the infected individuals that developed full blown aids. And assumed not be infective, so that we have,

$$N(t) = S(t) + I_1(t) + I_2(t) + A(t) \tag{1}$$

Considering susceptible population, it is increased by recruitment of individuals into the population at a rate b. the susceptible individuals may contracts the virus following contact with the infected individuals $(I_1(t) \text{and} I_2(t))$ at a rate $\lambda$ and it is given by

$$\lambda = (\beta_1 c_1 I_1 + \beta_2 c_2 I_2) \tag{2}$$

In the above equation $\beta_1$ and $\beta_2$ are the effective contact rates, i.e. contact capable of leading to infections. While $c_1$ and $c_2$ are the number of partners for $I_1$ and $I_2$ respectively. The susceptible population is reduced by natural death at a rate $(\varphi)$, so that the rate of change of susceptible population with respect to time is given by

$$\frac{ds}{dt} = b - (\beta_1 c_1 I_1 + \beta_2 c_2 I_2) - \varphi(s) \tag{3}$$

The population of asymptomatic class is generated by the infection of the susceptible individuals at a rate $\lambda(t)$. And the class is reduced by the natural death $(\varphi)$ as well as manifestation of the symptoms of the disease. Hence the rate of change of asymptomatic class with respect to the time is given by,

$$\frac{dI_1}{dt} = (\beta_1 c_1 I_1 + \beta_2 c_2 I_2)S - (\varphi + \alpha)I_1 \tag{4}$$

The population of symptomatic class is generated by the manifestation of the symptoms, of the disease by the asymptomatic individuals. And it is reduced by natural death and failure of treatments or development of resistant to the treatment. Thus, the rate of change of symptomatic class with respect to time is given by,

$$\frac{dI_2}{dt} = \alpha I_1 - (\tau + \varphi)I_2 \tag{5}$$

The population of Aids class is generated by the failure of the treatment or development of resistant to the treatment offered to the asymptomatic class, at a rate $(\tau)$ and the population is reduced through natural death as well as death due to infection $(\theta)$. Therefore the rate of change of Aids individuals with respect to time is

$$\frac{dA}{dt} = \tau I_2 - (\theta + \varphi)A \tag{6}$$

Then the model for the transmission dynamics of HIV/AIDS is given by the following non-linear system of differential equations

$$\begin{cases} \frac{dS}{dt} = b - (\beta_1 c_1 I_1 + \beta_2 c_2 I_2)S - \varphi S \\ \frac{dI_1}{dt} = (\beta_1 c_1 I_1 + \beta_2 c_2 I_2)S - (\varphi + \alpha)I_1 \\ \frac{dI_2}{dt} = \alpha I_1 - (\tau + \varphi)I_2 \\ \frac{dA}{dt} = \tau I_2 - (\theta + \varphi)A \end{cases} \tag{7}$$

From the system (7) above, it is assumed that not all infected individuals' take part in spreading the disease. As in the case of AIDS class, we assumed they are inactive and so they do not spread the virus.

## INVARIANT REGION

**Lemma:** The closed set $D = \{(S, I_1, I_2, A) \in \mathbb{R}_+^4 : N \le \frac{b}{\varphi}\}$ this is positively invariant and attracting with respect to model (7).

**Proof**: Adding all equations of model (7) it gives $\frac{dN}{dt} = b - \varphi N - \theta A \Rightarrow \frac{dN}{dt} = b - \varphi N$ since $\frac{dN}{dt} \le b - \varphi N$ then it follows that $\frac{dN}{dt} < 0$ if and only if $N(t) > \frac{b}{\varphi}$, therefore a standard comparison theorem can be used to show that $N(t) \le N(0)e^{-\varphi_t} + \frac{b}{\varphi}[1 - e^{-\varphi_t}]$ thus $N(t) \le \frac{b}{\varphi} \, if \, N(0) \le \frac{b}{\varphi}$ hence D is positively invariant in the population. Furthermore, if $N(t) > \frac{b}{\varphi}$ then either the solutions enters D in infinite time or $N(t)$ approaches $\frac{b}{\varphi}$ and all the variables $I_1, I_2, A$ approaches 0 hence D is attracting. That means all solutions in $\mathbb{R}$, eventually enters D, hence model system (7) is well posed epidemiologically and mathematically. Which means it is sufficient to study the dynamics of the model system (7) in D.

## POSITIVITY OF THE SOLUTION

Since our system is dealing with human population, all variables and parameters of the model are non-negative. Adding up the equation (7), we have

$$\frac{dN}{dt} = \frac{ds}{dt} + \frac{dI_1}{dt} + \frac{dI_2}{dt} + \frac{dA}{dt} \tag{8}$$

This will give us $\frac{dN}{dt} = b - \varphi N - \theta A$

**Lemma 1:** Let the initial data be $\{(s_0, I_{1_0}, I_{2_0}, A_0) \ge 0\} \in \Omega$: then the solution set $\{s(t), I_1(t), I_2(t), A(t)\}$ of our model system (7) is positive for all t > 0.

**Proof:** We prove the above lemma by considering the model equation (7). We now consider the first equation of model system (7), which is $\frac{ds}{dt} = b - \lambda s - \varphi s$

$$\frac{ds}{dt} = b - \lambda s - \varphi s \ge -(\lambda + \mu)s$$

$$\frac{ds}{dt} \geq -(\lambda+\varphi)s$$

Separating the variables and integrate the result gives,

$$\int \frac{ds}{s} \geq -\int (\lambda+\varphi)dt$$

$$s(t) > s(o)e^{(\lambda+\varphi)t} > o$$

If and only if $(\lambda+\varphi) > 0$

From the second equation of the model system (7)

$$\frac{dI_1}{dt} = \lambda s - (\alpha+\varphi)I_1$$

$$\frac{dI_1}{dt} \geq -(\alpha+\varphi)I_1$$

Separating the variables and integrate the result gives

$$\int \frac{dI_1}{s} \geq -\int (\alpha+\varphi)dt$$

$$I_1(t) > I_1(0)e^{-(\alpha+\varphi)t} > 0 \text{ If } (\alpha+\varphi) > 0$$

It follows for the rest of the equations of the model system (7). Therefore it is shown that the equations of the model system (7) are positive $\forall t > 0$.

## EXISTENCE OF STEADY STATES SOLUTION

We now analysed our model system (7) qualitatively in order to investigate the condition for the existence of equilibrium points. Let $E(s)$ $E(s^*, I_1^*, I_2^*, A^*)$ be the equilibrium points of our model system (7). The steady state solutions are obtained by equating the system (7) to zero and solve thus:

$$\begin{cases} b-(\beta_1 c_1 I_1+\beta_2 c_2 I_2)S-\varphi S=0 \\ (\beta_1 c_1 I_1+\beta_2 c_2 I_2)S-(\varphi+\alpha)I_1=0 \\ \alpha I_1-(\tau+\varphi)I_2=0 \\ \tau I_2-(\theta+\varphi)A=0 \end{cases} \tag{9}$$

So that $\frac{ds}{dt} = \frac{dI_1}{dt} = \frac{dI_2}{dt} = \frac{dA}{dt} = 0$.

## DISEASE FREE EQUILIBRIUM STATE

In the subsequent analysis, since variable A does not appear in the 1$^{st}$-3$^{rd}$ equations of the model system (7), we consider the first three equations as contained [19]. In as much as our recruitment rate (b) does not turn to zero, then our population will not go to the extinction that means there is no trivial equilibrium point. This implies that $(S^*, I_1^*, I_2^*) \neq (0,0,0)$. In absence of the disease, $(I_1^* = I_2^* = 0)$ thus, model system (7) is reduced to

$$\begin{cases} \frac{dS}{dt} = b - (\beta_1 c_1 I_1 + \beta_2 c_2 I_2)S - \varphi S \\ \frac{dI_1}{dt} = (\beta_1 c_1 I_1 + \beta_2 c_2 I_2)S - (\varphi - \alpha)I_1 \\ \frac{dI_2}{dt} = \alpha I_1 - (\tau + \varphi)I_2 \end{cases} \tag{10}$$

$$b - (\beta_1 c_1 I_1 + \beta_2 c_2 I_2)s - \varphi s + (\beta_1 c_1 I_1 + \beta_2 c_2 I_2)s - (\varphi + \alpha)I_1 + \alpha I_1 - (\tau + \varphi)I_2 = 0$$

Thus it reduced to $b - \varphi s = 0$, so that $s^* = \frac{b}{\varphi}$. Then our disease free equilibrium point is given by

$$E_0 = (\frac{b}{\varphi}, 0, 0) \tag{11}$$

## BASIC REPRODUCTION NUMBER

The basic reproduction number is the fundamental parameter that governed the spread or transmission of a disease. Hence, it is defined as the number of secondary infections generated by a typical infected individual in a disease free population throughout the period of its infectiousness [14-15]. To find the basic reproduction number $(R_0)$, we used the next generation operator method which is given by $[\frac{\partial F_i(E_0)}{\partial x_j}][\frac{\partial V_i(E_0)}{\partial x_j}]^{-1}$ using equation (10) above, F and V are, $F = \begin{bmatrix} (\beta_1 c_1 I_1 + \beta_2 c_2 I_2)s \\ 0 \\ 0 \end{bmatrix}$, $V = \begin{bmatrix} (\varphi + \alpha)I_1 \\ (\tau + \varphi)I_2 - \alpha I_1 \\ b + (\beta_1 c_1 I_1 + \beta_2 c_2 I_2)s + \varphi s \end{bmatrix}$ Jacobean matrices of F and V at the disease free equilibrium point $E_0$ are given as follows,

$$F(E_0) = \begin{bmatrix} \frac{\beta_1 c_1 b}{\varphi} & \frac{\beta_2 c_2 b}{\varphi} & 0 \\ 0 & 0 & 0 \\ 0 & 0 & 0 \end{bmatrix} = \begin{bmatrix} \frac{\beta_1 c_1 b}{\varphi} & \frac{\beta_2 c_2 b}{\varphi} \\ 0 & 0 \end{bmatrix}$$

$$V(E_0) = \begin{bmatrix} \varphi + \alpha & 0 & 0 \\ -\alpha & \tau + \varphi & 0 \\ 0 & 0 & 0 \end{bmatrix} = \begin{bmatrix} \varphi + \alpha & 0 \\ -\alpha & \tau + \varphi \end{bmatrix}$$

$$V^{-1} = \begin{bmatrix} \frac{1}{\varphi + \alpha} & 0 \\ \frac{\alpha}{(\varphi + \alpha)(\tau + \varphi)} & \frac{1}{\tau + \varphi} \end{bmatrix}$$

Thus,

$$R_0 = \frac{b[\beta_1 c_1 (\tau + \varphi) + \beta_2 c_2 \alpha]}{\varphi(\varphi + \alpha)(\tau + \varphi)} \tag{12}$$

Considering above, the basic reproduction number is a function of c, which is the number of sexual partners. In order to keep the spread of the disease at minimum, the number of sexual partners should be restricted.

## STABILITY OF DISEASE FREE EQUILIBRIUM

If all the Eigen values of the Jacobean matrix of the system (10) have negative real parts, then the disease free equilibrium is locally asymptotically stable, otherwise it is unstable. Therefore the Jacobi an of the system (10) at $E_0(\frac{b}{\varphi},0,0)$ takes the form

$$J(E_0)=\begin{vmatrix} -\varphi & -\frac{\beta_1 c_1 b}{\varphi} & -\frac{\beta_2 c_2 b}{\varphi} \\ 0 & -(\varphi+\alpha)+\frac{\beta_1 c_1 b}{\varphi} & \frac{\beta_2 c_2 b}{\varphi} \\ 0 & \alpha & -(\tau+\varphi) \end{vmatrix}$$

From above $trace(J)=-(\varphi+(\varphi+\alpha)+(\tau+\varphi))+\frac{\beta_1 c_1 b}{\varphi}$ it shows clearly that $trace(J)<0$ then for $\det(J)$ to be > 0, we proceed as follows by expanding det (J) we have $-\varphi\begin{vmatrix} -(\varphi+\alpha)+\frac{\beta_1 c_1 b}{\varphi} & \frac{\beta_2 c_2 b}{\varphi} \\ \alpha & -(\tau+\alpha) \end{vmatrix}$

After some algebraic simplification we got the relation as $\beta_1 c_1 b(\varphi+\tau)+\beta_2 c_2 b\alpha>\varphi(\varphi+\varphi\tau+\alpha\tau+\alpha\varphi)$ this means that $\frac{b[\beta_1 c_1(\tau+\varphi)+\beta_2 c_2\alpha]}{\varphi(\varphi+\alpha)(\tau+\varphi)}<1$.

This indicates that $R_0<1$ then this shows that disease free equilibrium is locally asymptotically stable otherwise it is unstable.

## ENDEMIC EQUILIBRIUM STATE

Let us consider the endemic equilibrium of our model system (10) such that $E^*(S^*,I_1^*,I_2^*)\neq(0,0,0)$ with

$$S^*=\frac{b}{\varphi R_0}\ I_1^*=\frac{b}{\mu+\delta}[1-\frac{1}{R_0}],\ I_2^*=\frac{1}{\mu+\delta}I_1^* \tag{13}$$

From the above equation (13), $I_1^*$ is positive if $R_0>0$ therefore $E^*(s^*,I_1^*,I_2^*)$ is the positive endemic equilibrium point that exist if $R_0>1$.

## LOCAL STABILITY OF ENDEMIC EQUILIBRIUM

We now obtain the Jacobean matrix of the model system (10) at the endemic equilibrium point i.e. $E^*(s^*,I_1^*,I_2^*)$ Thus it is given by

$$(J_{E^*})=\begin{vmatrix} -(\beta_1 c_1 I_1+\beta_2 c_2 I_2+\varphi) & -\beta_1 c_1 s & -\beta_2 c_2 s \\ \beta_1 c_1 I_1+\beta_2 c_2 I_2 & -(\varphi+\alpha)+\beta_1 c_1 s & \beta_2 c_2 s \\ 0 & \alpha & -(\tau+\varphi) \end{vmatrix}$$

All the state variables are at the endemic equilibrium point. Hence
$trace(J_{E^*})=-(\beta_1 c_1 I_1+\beta_2 c_2 I_2)+\beta_1 c_1 s-(3\varphi+\tau+\alpha)$ , clearly $trace(J_{E^*})<0$

We now find $\det(J_{E^*})$ to be > 0, we proceed as follows by expanding $\det(J_{E^*})$ and placing it to be > 0 we have $-\varphi^3+(\beta_1 c_1(s+I_1)-\alpha-\tau-\beta_2 c_2 I_2)\varphi^2+(2(\beta_2 c_2 I_2 s+\frac{\alpha I_1}{2})+\frac{\tau(s+I_1)}{2})\beta_1 c_1$ $+((s-I_2)\alpha-I_2\tau)\beta_2 c_2-\alpha\tau\varphi+2c_1\tau(\beta_2 c_2 I_2+\frac{\alpha I_1}{2})\beta_1+2\beta_2 c_2 I_2\alpha(\beta_2 c_2 s-\frac{\tau}{2})<1$ Clearly the above expression is < 1 this implies that the endemic equilibrium point $E^*(s^*,I_1^*,I_2^*)$, is locally asymptotically stable.

## OPTIMAL CONTROL FORMULATION

In this part, we need to consider time dependent controls. They are CD4+ counts and treatment of the infective, represented by $u_1$ and $u_2$ respectively, in order to curtail the spread of HIV/AIDS in a community. We therefore regard the entities $u_1$ and $u_2$ as function of time i.e. $u_1(t)$ and $u_2(t)$ . We now apply optimal control method in order to determine the necessary conditions for the control of HIV/AIDS. For us to investigate the optimal level of CD4+ counts and treatment for the infective that would be needed in order to control the disease. We then have an objective function J, which is to be minimised.

$$J = \int_0^{t_f} (D_1 I_1 + D_2 I_2 + D_3 A + D_4 u_1^2 + D_5 u_2^2) dt \tag{14}$$

Where $t_f$ is the final time and the coefficients $D_1, D_2, D_3, D_4$ and $D_5$ are the relative weight that help to balance each term, in the integrand so that no any of the term dominates. In the same vein, $D_1, D_2,$ and $D_3$ are the relative measures of the importance of reducing the associated classes on the spread of the disease. While $D_4$ and $D_5$ are the relative measures of the cost required to implement each of the associated controls. Our goal is to minimize the number of infected humans while minimizing the cost of control $u_1(t), u_2(t)$ thus we seek an optimal control $u_1^*, u_2^*$ such that $J(u_1^*, u_2^*) = \min_{u_1, u_2}\{J(u_1, u_2) | u_1, u_2 \in U$ where the control set

$$U = \{(u_1, u_2) | u_1 \text{ lebesque measurable}, 0 \le u_i \le u_{i\max} < 1, i = 1, 2\} \tag{15}$$

Hence, we now extend the model system (7) by introducing some control strategy to curtail the spread of HIV/AIDS. The control measures are CD4+ count and treatment for the infective, represented by $u_1$ and $u_2$ respectively. The importance of CD4+ count include, to verify when to start taking lifesaving treatment as well as weather the treatment is effective or not, The model system (7) becomes,

$$\begin{cases} \dfrac{ds}{dt} = b - (\beta_1 c_1 I_1 + \beta_2 c_2 I_2)s - \varphi s \\ \dfrac{dI_1}{dt} = (\beta_1 c_1 I_1 + \beta_2 c_2 I_2)s - (u_1 + \alpha + \varphi) I_1 \\ \dfrac{dI_2}{dt} = (\alpha + u_1) I_1 - (\tau + \varphi + u_2) I_2 \\ \dfrac{dA}{dt} = (\tau + u_2) I_2 - (\theta + \varphi) A \end{cases} \tag{16}$$

It now moved to determine the optimal combination of controls $u_1$ and $u_2$ that will be enough to minimize the cost of the CD4+ count, as well as the cost of the treatment for the infective, and at the same time to reduce the number of infective.

The necessary conditions, for an optimal control comes from pontryagin's maximum principle [18]. The principle convert equations (14) and (15) into a problem of minimizing point wise a Hamiltonian H, with respect to $u_1$ and $u_2$ we therefore characterize the optimal controls $u_1^*$ and $u_2^*$, which gives the optimal level for the two control measures and the corresponding states $(s^*, I_1^*, I_2^*, A)$ .

$$\begin{aligned} H = {} & D_1 I_1 + D_2 I_2 + D_3 A + D_4 u_1^2 + D_5 u_2^2 + \lambda_1 (b - (\beta_1 c_1 I_1 + \beta_2 c_2 I_2) S - \varphi S) \\ & + \lambda_2 ((\beta_1 c_1 I_1 + \beta_2 c_2 I_2) S - (u_2 + \varphi + \alpha) I_1) \\ & + \lambda_3 ((\alpha + u_1) I_1 - (u_2 + \tau + \varphi) I_2) \\ & + \lambda_4 ((\tau + u_2) I_2 - (\theta + \varphi + u_2) A) \end{aligned} \tag{17}$$

Where $\lambda_1, \lambda_2, \lambda_3$ and $\lambda_4$ are the adjoint variable.

**Theorem:** Let $(u_1^*, u_2^*) \in U$ be an optimal control with the corresponding states $(s^*, I_1^*, I_2^*, A)$ then there exist the ad joint variables $\lambda_i$ for $i = 1.......4$, satisfy

$$\begin{cases} \lambda_1' = (\beta_1 c_1 I_1 + \beta_2 c_2 I_2)(\lambda_1 - \lambda_2) + \lambda_1 \varphi \\ \lambda_2' = -D_1 + \beta_1 c_1 S(\lambda_1 - \lambda_2) + (\alpha + u_1)(\lambda_2 - \lambda_3) + \lambda_2 \varphi \\ \lambda_3' = -D_2 + \beta_2 c_2 S(\lambda_1 - \lambda_2) + (\tau + u_2)(\lambda_3 - \lambda_4) + \lambda_3 \varphi \\ \lambda_4' = -D_3 + \lambda_4 (\theta + \varphi) \end{cases} \tag{18}$$

The transversality conditions is $\lambda_i(t_f)=0$, for $i=1......4$ with the optimal control defined as

$$u_1^* = \min\{\max(0, \frac{(\lambda_2-\lambda_3)I_1}{2D_4}).u_{1\max}\}.$$

$$u_2^* = \min\{\max(0, \frac{(\lambda_3-\lambda_4)I_2}{2D_5}).u_{1\max}\}.$$

**Proof:** We used pontryagin's maximum principle to obtain the differential equation governing the adjoint variables as follows,

$$\begin{cases} \lambda_1^{'} = -\frac{\partial H}{\partial S} = (\beta_1 c_1 I_1 + \beta_2 c_2 I_2)(\lambda_1 - \lambda_2) + \lambda_1\varphi \\ \lambda_2^{'} = -\frac{\partial H}{\partial I_1} = -D_1 + \beta_1 c_1 S(\lambda_1 - \lambda_2) + (\alpha + u_1)(\lambda_2 - \lambda_3) + \lambda_2\varphi \\ \lambda_3^{'} = -\frac{\partial H}{\partial I_2} = -D_2 + \beta_2 c_2 S(\lambda_1 - \lambda_2) + (\tau + u_2)(\lambda_3 - \lambda_4) + \lambda_3\varphi \\ \lambda_4^{'} = -\frac{\partial H}{\partial A} = -D_3 + \lambda_4(\theta + \varphi) \end{cases} \quad (19)$$

The Hamiltonian is maximized with respect to the controls at the optimal control $u^* = (u_1^*, u_2^*)$, then we differentiate H with respect to $u_1$ and $u_2$ on U respectively, hence we obtained,

$$\begin{cases} \frac{\partial H}{\partial u_1} = 2D_4 u_1 - \lambda_2 I_1 + \lambda_3 I_1 \\ \frac{\partial H}{\partial u_2} = 2D_5 u_2 - \lambda_3 I_2 + \lambda_4 I_2 \end{cases} \quad (20)$$

We then imposed the bounds on our controls that is $0 \le u_1 \le u_{1\max}$, $0 \le u_2 \le u_{2\max}$ and we obtained

$$\begin{cases} u_1^* = \min\{\max(0, \frac{(\lambda_2-\lambda_4)I_1}{2D_4})u_{1\max}\} \\ u_2^* = \min\{\max(0, \frac{(\lambda_3-\lambda_4)I_2}{2D_5})u_{2\max} \end{cases} \quad (21)$$

When we differentiate the Hamiltonian function for the second time, with respect to $u_1$ and $u_2$ it yields

$$\begin{cases} \frac{\partial^2 H}{\partial u_1^2} = 2D_4 \\ \frac{\partial^2 H}{\partial u_2^2} = 2D_5 \end{cases} \quad (22)$$

The second partial derivative of (20) above with respect to $u_1$ and $u_2$ respectively are positive, this implies that the optimal problem is minimum at $u_1$ and $u_2$ .

## NUMERICAL SIMULATION

In this section, we examined the extended model with two optimal controls, which is CD4+ count and treatment for the infective. Numerical effect of such controls on the spread of HIV/AIDS in a population is investigated by the use of Mathematica and presented in TABLE 1, FIGURE 2 and FIGURE 3.

**TABLE 1.** Describe the parameters used in model equation (7).

| Parameter | Value | Source |
|---|---|---|
| b | 3.1 | estimated |
| $\beta_1$ | 0.11 | [16] |
| $\beta_2$ | 0.55 | [16] |
| $c_1$ | 0.43 | estimated |
| $c_2$ | 0.32 | estimated |
| $\varphi$ | 0.02 | [17] |
| $\theta$ | 0.70 | estimated |
| $\alpha$ | 0.23 | estimated |
| $\tau$ | 0.17 | estimated |
| $u_1$ | 0.2 | [16] |
| $u_2$ | 0.5 | [16] |

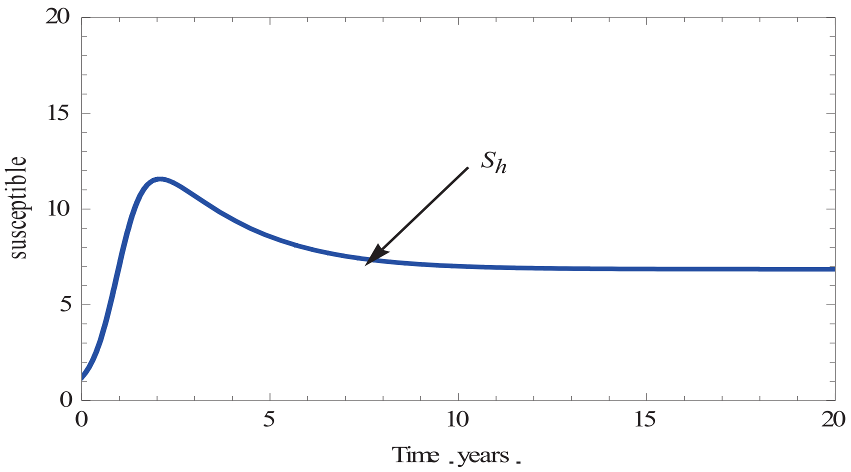

**FIGURE 2**. The susceptible individuals.

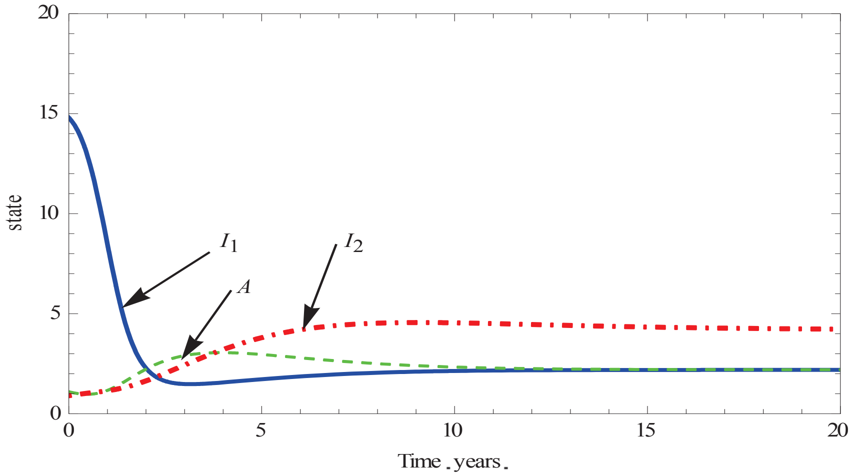

**FIGURE 3.** The infected classes.

With the CD4+ count of the infected human $u_1$ and treatment for the infective $u_2$ both of them are used to optimize the objective function J. We observed that the control strategy yields increase in the life span of the infected human, this indicate the importance of CD4+ count and treatment.

## CONCLUSION

In this paper, we developed a mathematical model for the spread of HIV/AIDS in a population. Invariant region, positivity and local stability are investigated; the model analysis shows that the disease free equilibrium is locally asymptotically stable whenever the threshold parameter $R_0$ is less than 1, i.e. $R_0 < 1$, unstable otherwise. The existence of optimal control is established analytically by the use of optimal control theory; the optimal control theory has a very desirable effect in reducing both the infected human populations there by increasing the population of susceptible class. From our simulation result, it was found that both the cd4+ counts as well as treatment for the infective will help in reducing the spread of the disease and increasing the life span of the infected, and hence delaying the onset of AIDS.

## ACKNOWLEDGMENTS

The present work is supported by short term grant (PMATHS/6313040) from RCMO, Universiti Sains Malaysia. We also like to express our sincere thanks to School of Mathematical Sciences for providing adequate facilities for the research.
